# Network pharmacology and *in vitro* experimental verification to explore the mechanism of Sanhua decoction in the treatment of ischaemic stroke

**DOI:** 10.1080/13880209.2021.2019281

**Published:** 2022-01-05

**Authors:** Wei Zhang, Li Zhang, Wen jun Wang, Shanbo Ma, Mingming Wang, Minna Yao, Ruili Li, Wei wei Li, Xian Zhao, Dongmei Hu, Yi Ding, Jingwen Wang

**Affiliations:** a Department of Pharmacy, Xijing Hospital, Fourth Military Medical University, Xi'an, Shaanxi, China; b Department of Emergency, Xijing Hospital, Fourth Military Medical University, Xi'an, Shaanxi, China

**Keywords:** Cerebral ischaemia, cell viability, SH-SY5Y cells, molecular docking

## Abstract

**Context:**

Stroke is an illness with high morbidity, disability and mortality that presents a major clinical challenge. Sanhua decoction (SHD) has been widely used to treat ischaemic stroke in the clinic. However, the potential mechanism of SHD remains unknown.

**Objective:**

To elucidate the multitarget mechanism of SHD in ischaemic stroke through network pharmacology and bioinformatics analyses.

**Materials and methods:**

Network pharmacology and experimental validation approach was used to investigate the bioactive ingredients, critical targets and potential mechanisms of SHD against ischaemic stroke. Four herbal names of SHD, ‘ischemic stroke’ or ‘stroke’ was used as a keyword to search the relevant databases. SH-SY5Y cells were treated with various concentrations of SHD (12.5, 25, 50 or 100 μg/mL) for 4 h, exposed to oxygen and glucose deprivation (OGD) for 1 h, then reoxygenation for 24 h. The cell viability was detected by MTT, the lactate dehydrogenase (LDH) was evaluated by ELISA, and protein expression was detected by western blots.

**Results:**

SHD treatment increased the survival rate from 65.9 ± 4.3 to 85.56 ± 5.7%. The median effective dose (ED_50_) was 47.1 μg/mL, the LDH decreased from 288.0 ± 12.0 to 122.8 ± 9.1 U/L and the cell apoptosis rate decreased from 33.6 ± 1.8 to 16.3 ± 1.2%. Western blot analysis revealed that SHD increased the levels of p-PI3k, p-Akt and p-CREB1, and decreased the expression of TNF-α and IL-6.

**Discussion and conclusions:**

This study suggests that SHD protects against cerebral ischaemic injury via regulation of the PI3K/Akt/CREB1 and TNF pathways.

## Introduction

The disease burden of ischaemic stroke is increasing worldwide (Hankey 2017). The situation is especially serious in China. According to one report, stroke-related deaths in China account for nearly a third of the global total (Wang et al. 2017). Tissue plasminogen activators are currently the only effective method for stroke treatment. However, due to the narrow treatment window and easy bleeding, few patients truly benefit from such treatment (Rangaraju et al. 2015). The development of new safe and effective drugs for cerebral apoplexy is urgently needed.

Traditional Chinese medicines (TCMs) have shown significant effects in treating ischaemic stroke and have been used in the clinic for many years (Li et al. 2016; Seto et al. 2016). The active ingredients in TCM herbs treat ischaemic stroke through various biological processes, such as antioxidation, neuroinflammation and autophagy (Ding et al. 2014; Guo et al. 2015; Chen et al. 2019). Sanhua decoction (SHD) contains *Magnolia officinalis* (MO; Pinyin name Hou Pu; Scientific name *Magnolia officinalis* Rehd. et Wils [Magnoliaceae]), the young fruit of *Fructus aurantii immaturus* (FA; Pinyin name Zhi Shi; Scientific name *Citrus aurantium* (L.) [Rutaceae]), the root of *Rhei Radix et Rhizoma* (RR; Pinyin name Da Huang; Scientific name *Rheum officinale* Baill [Polygonaceae]) and *Notopterygii Rhizoma* (NR; Pinyin name Qiang Huo; Scientific name *Notopterygium incisum* Ting ex H. T. Chang [Apiaceae]) in a ratio of 4:2:2:1.5 (w/w). Based on TCM theory, SHD was used to soothe Qi flow and dredge sweat pores (Fan et al. 2012). Clinically, SHD is extensively used to manage ischaemic stroke, and it has been especially effective in reducing blood viscosity and regulating circulation disorders (Yang et al. 2009; Liu 2011). In addition, the anthraquinone constituent of RR and the honokiol of MO have protective effects against ischaemic stroke (Liou et al. 2003; Li et al. 2019). Recent studies have revealed that SHD downregulates the expression of AQP4 and p-tau, promotes neurological function and reduces cerebral infarction (Lin et al. 2015; Fu et al. 2020). However, the material basis of SHD and the potential mechanism of its anti-ischaemic stroke activity remain unclear.

**Figure 1.**
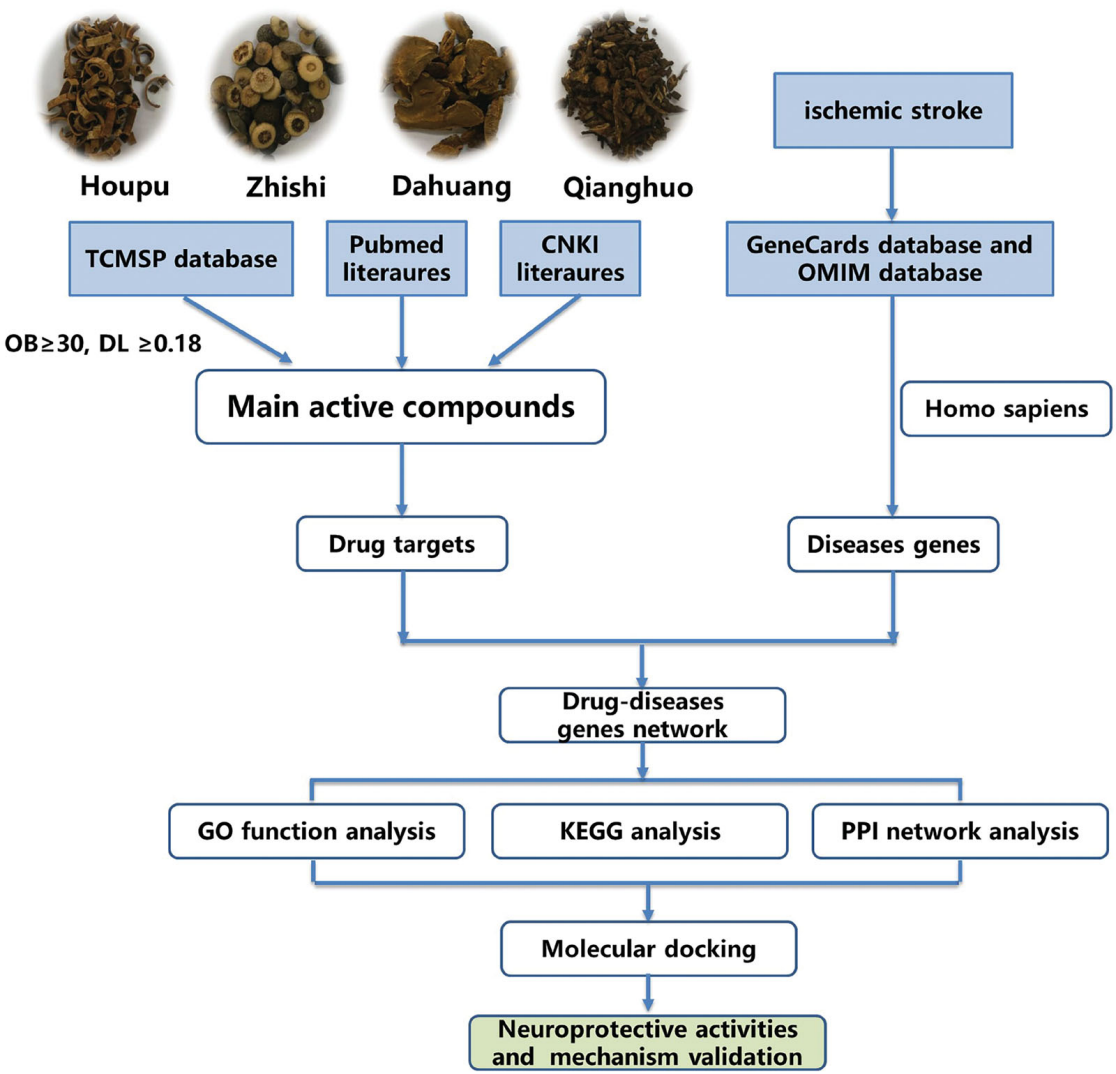
The work flow to elucidate the mechanism of SHD.

Network pharmacology provides new methods for studying the complex pharmacological mechanisms of Chinese herbs (Zhu et al. 2018). This technique has been used to study multitarget drug therapy and mechanisms of drug action. Based on the data analysis, network pharmacology could comprehensively investigate the pharmacological activities and mechanisms of TCM components and is suitable for the study of complex proprietary Chinese medicines (Xu et al. 2017). In this study, the mechanism by which SHD treats ischaemic stroke was studied by using network pharmacology methods. The mechanism of SHD suggested by network analysis was confirmed by pharmacological analysis. A chart of the workflow to elucidate the mechanism of action of SHD in the treatment of ischaemic stroke is shown in Figure 1.

## Materials and methods

### Database construction

The composition of SHD was obtained from the TCMSP2.3 database and duplicate data were removed (Ru et al. 2014), providing comprehensive information on herbal components, such as chemical structure and oral bioavailability (OB), drug-likeness (DL) and drug targets. The pharmacokinetic parameters OB and DL are important contributors to bioactivity. The active ingredients were selected with OB ≥30% and DL ≥0.18 (Xu et al. 2012; Liu et al. 2013). Compound-related targets were screened from TCMSP 2.3 and Swiss Target Prediction (http://www.swisstargetprediction.ch/) by limiting to ‘*Homo sapiens*’. The formal gene name and UniProt ID of all genes were switched from UniProt. The disease-related targets were assembled from the OMIM (updated 30 July 2021) and GeneCards 5.4 databases.

### Network construction and analysis

The biological networks were analysed and visualized by Cytoscape 3.7.1 software (Shannon et al. 2003). The Venn diagram tool (http://bioinformatics.psb.ugent.be/webtools/Venn/) was used to establish SHD component-related targets and their overlap with ischaemic stroke-related targets. To construct the interaction network between SHD components and the targets of ischaemic stroke, a network analyser was used to calculate degree centrality (DC), medium number centrality (BC) and tight centrality (CC) in Cytoscape to identify the core nodes in the interactive network. These parameters are predefined and used for network analysis (Jiang et al. 2019).

### Protein–protein interaction data

The protein interaction was confirmed by the STRING database (Szklarczyk et al. 2017). The PPI data were searched using version 11.0 of STRING with the following search settings: restricted to ‘*Homo sapiens*’. Ten targets with the highest link as the key genes for ischaemic stroke were identified.

### GO and KEGG enrichment analysis

Gene ontology (GO) data and Kyoto Encyclopedia of Genes and Genomics (KEGG) pathway were performed (Huang et al. 2009) based on integrated visualization and comments from DAVID 6.8 database. The top 20 categories were selected for following studies.

### Molecular docking

AutoDock Vina 1.1.2 software (Trott and Olson 2010) was used to pre-process the receptors and ligands and perform molecular docking. We used Drugbank 5.1.8 to identify drugs positive for the core targets (Jia et al. 2021). First, the 3D and 2D structures of core components and positive drugs as ligands were downloaded from PubChem, and stored in a PDB file format for 3D and SDF file format for 2D. The crystal structures of key proteins obtained from the PPI analysis were searched from the RCSB v1.0 database. Next, the compounds and target proteins were imported into the software as requested. The docking simulation was performed under the AutoDock 4.2 program. The results were analysed and visualized using Discovery Studio Visualizer 4.0 (Zhang et al. 2021).

### Pharmacological verification of network analysis

#### Chemicals and reagents

MO, FA, RR and NR in SHD were purchased from the pharmacy of Xijing Hospital, and all were included in the Chinese Pharmacopoeia. The plant materials were authenticated by Professor Haifeng Tang. Voucher specimen (20C266, 20C315, 20C364, 20C375) was preserved in the herbarium of Xijing Hospital. SHD extract was prepared according to the following experimental steps: first, the essential oil was extracted, boiled in water and extracted three times. Then, the extracting solution was filtered and condensed by rotary evaporation. Finally, the solution was concentrated to 1 g/mL. The original solution was stored in a 4 °C refrigerator. The quality control of SHD is shown in Supplementary materials. The anti-PI3k, p-PI3k, Akt, p-Akt, CREB1, p-CREB1, TNF-α, IL-6 and β-actin for Western blotting were purchased from Cell Signaling Technology (Danvers, MA).

### Cell culture and treatment

Neuroblastoma SH-SY5Y cells were obtained from American Type Culture Collection (Manassas, VA). SH-SY5Y cells were maintained in DMEM/Ham’s F12 1:1 medium supplemented with 10% (v/v) FBS, 100 U/mL penicillin and streptomycin. The cells were grown at 37 °C with 5% CO_2_ and 95% humidity. All *in vitro* experiments were performed 24 h after cell culture, and the cells were inoculated onto plates at specific densities. The cells were treated with different concentrations of SHD extract (12.5, 25, 50 or 100 μg/mL, *n* = 6) for 4 h before oxygen and glucose deprivation (OGD). Then, to establish an OGD model *in vitro*, the cells were exposed to a gas mixture of 95% N_2_/5% CO_2_ at 37 °C for 1 h. After OGD, the cells were transferred to an incubator with reoxygenation and normal medium for 24 h. Control cells (*n* = 6) were incubated under normal culture conditions for the same period.

### Cell viability analysis

The MTT assay was used to evaluate the cell viability. The cells were cultured at 1 × 10^4^ cells per well in 96-well plates. Then, 5 mg/mL MTT was added to normoxia-conditioned medium and incubated at 37 °C for 4 h according to the experimental protocol. The optical density (OD) was measured at 490 nm. Cell damage was observed by lactate dehydrogenase (LDH) activity in the injured cells. LDH release was evaluated by an ELISA kit based on the specified method.

### Flow cytometry for cell apoptosis analysis

Cell apoptosis was detected by assessing Annexin V-FITC and propidium iodide (PI) uptake. In brief, SH-SY5Y cells were washed with 1 × annexin V-FITC binding buffer and incubated with annexin V-FITC and PI for 15 min at room temperature in the dark, and then apoptotic cells were subjected to flow cytometry analysis.

### Western blot analysis

Cells were lysed using lysis buffer, homogenized and centrifuged, and the supernatant was collected. The protein concentration was evaluated by the Bradford method (Kielkopf et al. 2020). The proteins were isolated by SDS-PAGE and transferred to PVDF membranes. The membranes were incubated at 4 °C overnight with a suitable primary antibody. Next, the membrane was incubated with anti-rabbit or anti-mouse IgG secondary antibody for 1 h. The blots were visualized with an ECL chemiluminescent kit (Millipore, Billerica, MA) and detected by a Western blotting system.

### Statistical analysis

Data were expressed as the mean ± standard deviation. Statistical significance was determined using one-way ANOVA followed by Tukey’s comparison tests for comparisons between two groups. *p* < 0.05 was defined as significant.

## Results

### Screening for SHD active ingredients

A total of 481 active compounds were collected in SHD from the database. Among these components, 139 active compounds were from MO, 65 active compounds were from FA, 92 active compounds were from RR and 185 active compounds were from NR. The ADME model and literature were combined to confirm the identification of active compounds.

A total of 57 potential compounds were obtained by ADME screening. Furthermore, among the compounds that did not meet the screening requirements, 12 compounds (honokiol and magnolol from MO (Huang et al. 2018), hesperidin (Oztanir et al. 2014), tangeretin (Wu et al. 2019), naringin (Yang et al. 2020) and apigenin (Nabavi et al. 2018) from FA, and physcion (Zhang et al. 2005), emodin, chrysophanol (Su et al. 2020), aloe-emodin, aloe-emodin-omega-*O*-β-d-glucopyranoside and emodin-8-*O*-β-d-glucopyranoside from RR) were also included in this study due to their significant biological activity based on the previous literature. After eliminating duplicates, 67 active compounds were defined from SHD (Table 1).

**Table 1.** The active compounds among the SHD for network analysis.

No.	Herb	Mol ID	Molecule name	CAS	MW	OB (%)	DL
1	AF	MOL007879	Tetramethoxyluteolin	855-97-0	342.37	43.68	0.37
2	AF	MOL005814	Tangeretin	481-53-8	372.4	21.38	0.43
3	AF	MOL001803	Sinensetin	2306-27-6	372.4	50.56	0.45
4	AF	MOL013433	Prangenin hydrate	31575-93-6	304.32	72.63	0.29
5	AF	MOL013430	Prangenin	2880-49-1	286.3	43.6	0.29
6	AF	MOL013276	Poncirin	14941-08-3	594.62	36.55	0.74
7	AF	MOL013435	Poncimarin	55916-48-8	330.41	63.62	0.35
8	AF	MOL013352	Obacunone	751-03-1	454.56	43.29	0.77
9	AF	MOL005828	Nobiletin	478-01-3	402.43	61.67	0.52
10	AF	MOL001798	Neohesperidin_qt	13241-33-3	302.3	71.17	0.27
11	AF	MOL005812	Naringin	10236-47-2	580.59	6.92	0.78
12	AF	MOL004328	Naringenin	480-41-1	272.27	59.29	0.21
13	AF	MOL000006	Luteolin	491-70-3	286.25	36.16	0.25
14	AF	MOL013277	Isosinensetin	17290-70-9	372.4	51.15	0.44
15	AF	MOL013428	Isosakuranetin-7-rutinoside	14259-47-3	594.62	41.24	0.72
16	AF	MOL013436	Isoponcimarin	59176-65-7	330.41	63.28	0.31
17	AF	MOL007930	Hesperidin	520-26-3	610.62	13.33	0.67
18	AF	MOL002914	Eriodyctiol (flavanone)	487-26-3	288.27	41.35	0.24
19	AF	MOL005849	Didymin	14259-47-3	286.3	38.55	0.24
20	AF	MOL013440	Citrusin B	105279-10-5	568.63	40.8	0.71
21	AF	MOL000008	Apigenin	520-36-5	270.25	23.06	0.21
22	AF, NR	MOL001941	Ammidin	482-44-0	270.3	34.55	0.22
23	AF	MOL013437	6-Methoxy aurapten	28587-44-2	328.44	31.24	0.3
24	AF	MOL005100	5,7-Dihydroxy-2-(3-hydroxy-4- methoxyphenyl)chroman-4-one	520-33-2	302.3	47.74	0.27
25	AF	MOL013279	5,7,4′-Trimethylapigenin	5631-70-9	312.34	39.83	0.3
26	AF	MOL009053	4-[(2S,3R)-5-[(E)-3-hydroxyprop-1-enyl]- 7-methoxy-3-methylol-2,3-dihydrobenzofuran- 2-yl]-2-methoxy-phenol	4263-87-0	358.42	50.76	0.39
27	NR	MOL000359	Sitosterol	64997-52-0	414.79	36.91	0.75
28	NR	MOL002644	Phellopterin	2543-94-4	300.33	40.19	0.28
29	NR	MOL011975	Notoptol	88206-49-9	354.43	62.97	0.48
30	NR	MOL011974	Notopterol	8206-46-6	352.46	8.71	0.46
31	NR	MOL004792	Nodakenin	495-31-8	408.44	57.12	0.69
32	NR	MOL001942	Isoimperatorin	482-45-1	270.3	45.46	0.23
33	NR	MOL011971	Diversoside_qt	5062-36-7	332.43	67.57	0.31
34	NR	MOL002881	Diosmetin	520-34-3	300.28	31.14	0.27
35	NR	MOL011969	Demethylfuropinnarin	N/A	270.3	41.31	0.21
36	NR	MOL011968	Coumarin, glycoside	N/A	534.61	33.07	0.78
37	NR	MOL001956	Cnidilin	14348-22-2	300.33	32.69	0.28
39	NR	MOL001951	Bergaptin	7380-40-7	338.43	41.73	0.42
40	NR	MOL011963	8-Geranoxy-5-ethoxypsoralen	N/A	368.46	40.97	0.5
41	NR	MOL011962	6′-Feruloylnodakenin	131623-14-8	584.62	32.02	0.67
42	MO	MOL005980	Neohesperidin	13241-33-3	302.3	57.44	0.27
43	MO	MOL000210	Magnolol	528-43-8	266.36	69.19	0.15
44	MO	MOL005955	Honokiol	35354-74-6	266.36	60.67	0.15
45	MO	MOL005970	Eucalyptol	470-82-6	266.36	60.62	0.32
46	RR	MOL002281	Toralactone	41743-74-2	272.27	46.46	0.24
47	RR	MOL002280	Torachrysone-8-*O*-beta-d-(6′-oxayl)-glucoside	N/A	480.46	43.02	0.74
48	RR	MOL002276	Sennoside E_qt	11137-63-6	524.5	50.69	0.61
49	RR	MOL002293	Sennoside D_qt	37271-17-3	524.5	61.06	0.61
50	RR	MOL002268	Rhein	478-43-3	284.23	47.07	0.28
51	RR	MOL002260	Procyanidin B-5,3′-*O*-gallate	106533-60-2	730.67	31.99	0.32
52	RR	MOL002259	Physciondiglucoside	84268-38-2	608.6	41.65	0.63
53	RR	MOL002257	Physcion-8-*O*-beta-d-glucopyranoside	75721-38-9	462.44	8.2	0.85
54	RR	MOL000476	Physcion	521-61-9	284.28	22.29	0.27
55	RR	MOL002303	Palmidin A	17062-55-4	510.52	32.45	0.65
56	RR	MOL002251	Mutatochrome	515-06-0	552.96	48.64	0.61
57	RR	MOL000554	Gallic acid-3-*O*-(6′-*O*-galloyl)-glucoside	N/A	484.4	30.25	0.67
58	RR	MOL002235	Eupatin	19587-65-6	360.34	50.8	0.41
59	RR	MOL003353	Emodinanthrone	491-60-1	256.27	24.72	0.21
60	RR	MOL002288	Emodin-1-*O*-beta-d-glucopyranoside	38840-23-2	432.41	44.81	0.8
61	RR	MOL000472	Emodin	518-82-1	270.25	24.4	0.24
62	RR	MOL002297	Daucosterol_qt	474-58-8	386.73	35.89	0.7
63	RR	MOL001729	Chrysophanol	481-74-3	254.25	18.64	0.21
64	RR	MOL002241	Aloe-emodin-ω-*O*-β-d-glucopyranoside	29010-56-8	432.41	9.04	0.81
65	RR	MOL000471	Aloe-emodin	481-72-1	270.25	83.38	0.24
66	RR	MOL002298	Aloe-emodin	481-72-1	272.27	20.65	0.24
67	RR	MOL000096	(–)-Catechin	7295-85-4	290.29	49.68	0.24

### SHD compound-target network analysis

**Figure 2.**
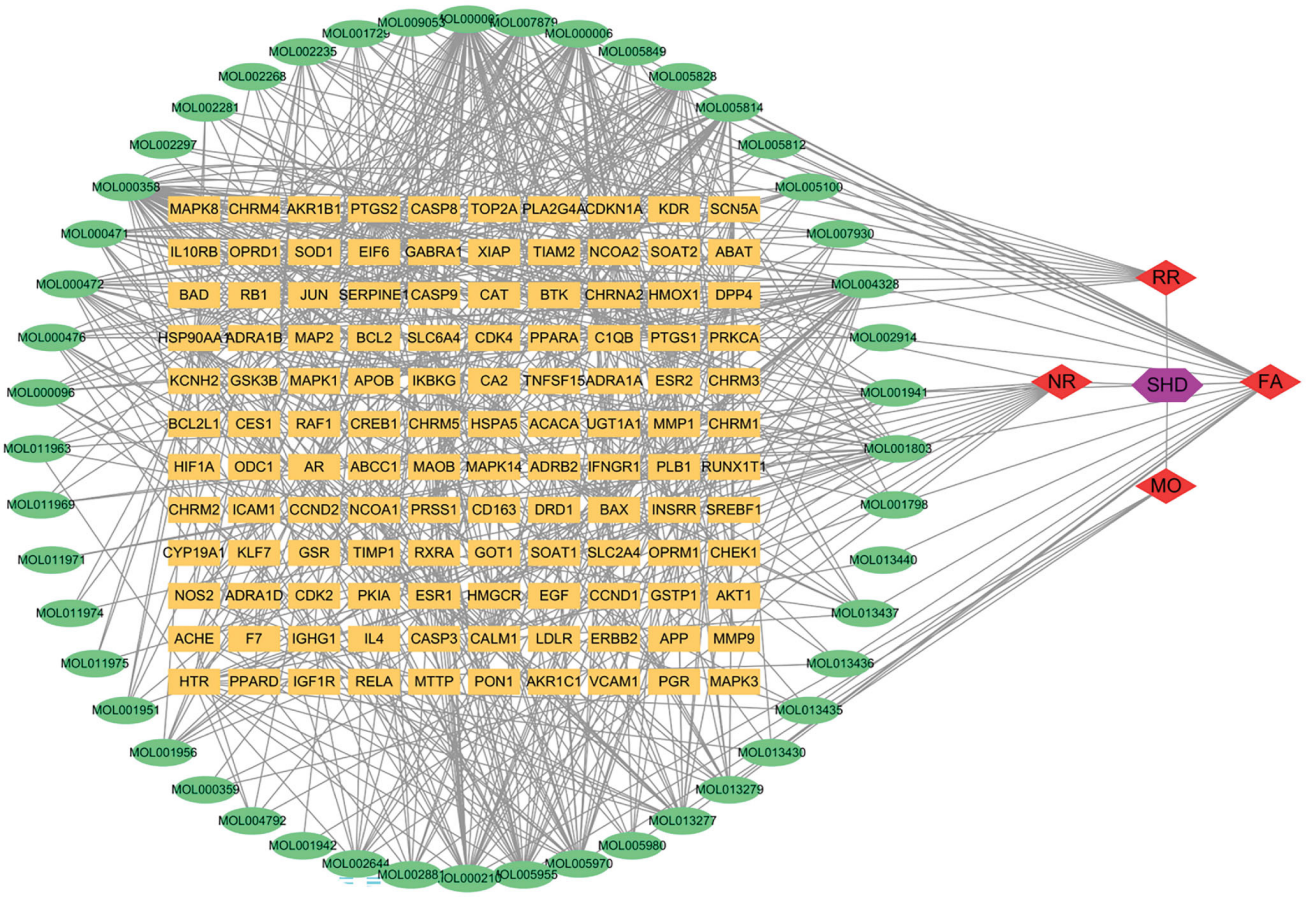
The compound-target network of SHD and analysis. The orange rectangles represent the potential targets, the green circles represent the compounds, the red diamonds represent the herbs and the purple hexagon represents SHD.

A total of 685 ingredients related targets were defined from the databases. To clarify the relationships among the herbs, an herb-constituent-target network of SHD was constructed, and the results are shown in Figure 2. The network consists of 235 nodes and 685 edges. Apigenin, β-sitosterol, luteolin, naringenin, nobiletin, honokiol, emodin, tetramethoxyluteolin, magnolol, tangeretin, eucalyptol, isosinensetin, aloe-emodin and sinensetin were predicted to be important active compounds in SHD by degree and intermediate centrality analyses.

### Potential SHD targets treat ischaemic stroke and network analysis

**Figure 3.**
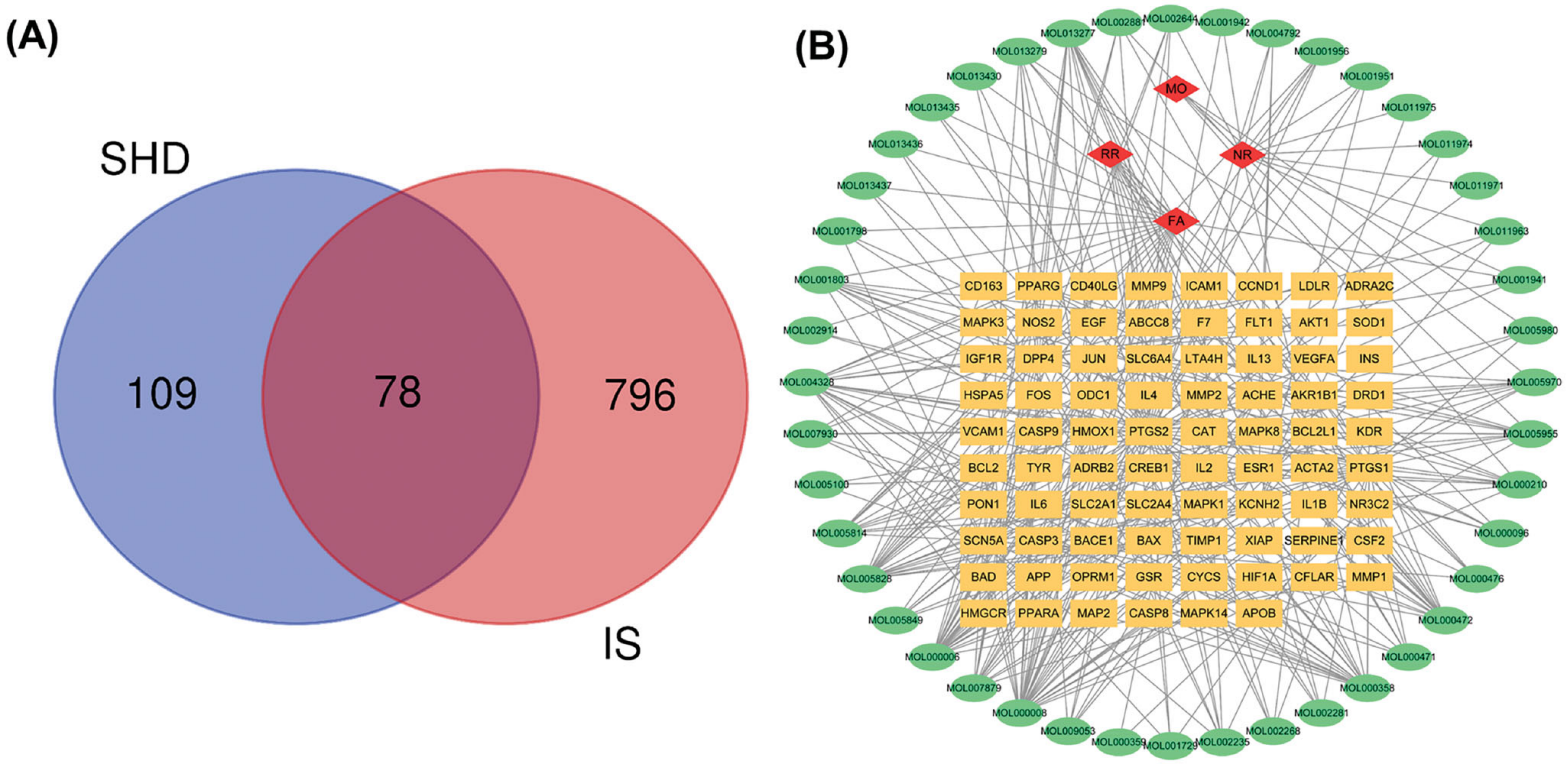
Venn diagram and disease-compound-target network of SHD. (A) The intersection of the potential SHD and ischaemic stroke targets. (B) Network of the targets shared between SHD and ischaemic stroke. The orange rectangles represent the potential targets, the green circles represent the compounds and the red diamonds represent the herbs.

To elucidate the mechanism and pharmacodynamics of SHD, we assembled ischaemic stroke-related genes from the GeneCards and OMIM databases, which contain publicly available microarray data. A total of 874 genes relevant to ischaemic stroke were identified from the gene databases. Further analysis with Venn diagrams identified 78 targets associated with both ischaemic stroke and SHD that are displayed in Figure 3(A). The potential gene-related ingredients are listed in Table 2. The disease-ingredient-target network was established based on the strength of the 78 shared targets identified as both compound targets and ischaemic stroke targets. Figure 3(B) displays those 126 nodes and 340 edges in this interaction network. Among the compounds that can interact with ischaemic stroke-related targets, apigenin, luteolin, nobiletin, naringenin, β-sitosterol, emodin, tetramethoxyluteolin, isosinensetin and tangeretin were connected with more than 10 genes. Moreover, the genes encoding PTGS2, PTGS1, SCN5A, DPP4, ADRB2, ESR1, KCNH2, F7, NOS2, PPARG, ACHE, CAS, P3, JUN and BAX were connected to more than five ingredients. The disease-compound-target network demonstrated the comprehensive regulation characteristics of multiple ingredients and multiple targets of SHD.

**Table 2.** The potential target genes associated compounds.

No.	Gene	Compound
1	XIAP	MOL000008, MOL000006
2	VEGFA	MOL000008, MOL000006
3	VCAM1	MOL007930
4	TYR	MOL000006
5	TIMP1	MOL005828
6	SOD1	MOL004328
7	SLC6A4	MOL005970, MOL000210, MOL000358, MOL005955
8	SLC2A4	MOL000472, MOL000006, MOL000008
9	SLC2A1	MOL000472
10	SERPINE1	MOL000008
11	SCN5A	MOL013279, MOL013277, MOL007879, MOL005980, MOL005970, MOL005849, MOL005828, MOL005814, MOL005100, MOL002644, MOL002235, MOL001956, MOL001951, MOL001803, MOL001798, MOL001729, MOL000476, MOL000358, MOL000210, MOL000008
12	PTGS2	MOL013437, MOL013436, MOL013435, MOL013430, MOL013279, MOL013277, MOL011974, MOL011963, MOL009053, MOL007930, MOL007879, MOL005980, MOL005970, MOL005955, MOL005849, MOL005828, MOL005814, MOL005100, MOL004792, MOL002914, MOL004328, MOL002881, MOL002644, MOL002281, MOL002268, MOL002235, MOL001956, MOL001951, MOL001942, MOL001941, MOL001803, MOL001798, MOL001729, MOL000476, MOL000472, MOL000471, MOL000358, MOL000210, MOL000096, MOL000008, MOL000006
13	PTGS1	MOL013279, MOL013277, MOL007879, MOL005980, MOL005970, MOL005955, MOL005849, MOL005828, MOL005814, MOL005100, MOL004328, MOL002914, MOL002881, MOL002281, MOL002268, MOL001803, MOL001798, MOL001729, MOL000476, MOL000471, MOL000472, MOL000358, MOL000210, MOL000096, MOL000008, MOL000006
14	PPARG	MOL013277, MOL007879, MOL005955, MOL005828, MOL004328, MOL000472, MOL000210, MOL000096, MOL000006
15	PPARA	MOL004328
16	PON1	MOL000358
17	OPRM1	MOL005970, MOL000358
18	ODC1	MOL000008
19	NR3C2	MOL000359
20	NOS2	MOL013279, MOL013277, MOL007879, MOL005828, MOL005814, MOL002881, MOL002281, MOL002235, MOL001803
21	MMP9	MOL005828, MOL000472, MOL000008, MOL000006
22	MMP2	MOL000006
23	MMP1	MOL000008, MOL000472, MOL000006
24	MAPK8	MOL005828
25	MAPK3	MOL004328
26	MAPK14	MOL007879, MOL005955, MOL000210
27	MAPK1	MOL004328, MOL000006
28	MAP2	MOL000358
29	LTA4H	MOL005955
30	LDLR	MOL004328
31	KDR	MOL002235, MOL000472
32	KCNH2	MOL013277, MOL011975, MOL011974, MOL011963, MOL009053, MOL007879, MOL005828, MOL005814, MOL001951, MOL001803, MOL000358
33	JUN	MOL005828, MOL002268, MOL000358, MOL000008, MOL000006
34	INS	MOL000008
35	IL6	MOL000006
36	IL4	MOL000008, MOL000006
37	IL2	MOL000008, MOL000006
38	IL1B	MOL000472, MOL000471
39	IL13	MOL000008
40	IGF1R	MOL000008
41	ICAM1	MOL000008, MOL007930, MOL000006
42	HSPA5	MOL005814
43	HMOX1	MOL005814, MOL000008, MOL000006
44	HMGCR	MOL004328
45	HIF1A	MOL000008
46	GSR	MOL004328
47	FOS	MOL000008
48	FLT1	MOL000472
49	F7	MOL013277, MOL007879, MOL005828, MOL005814, MOL002235, MOL001803, MOL000476, MOL000472, MOL000008
50	ESR1	MOL013435, MOL013277, MOL009053, MOL005955, MOL005849, MOL005828, MOL004792, MOL004328, MOL002281, MOL000210, MOL000096
51	EGF	MOL000472
52	DRD1	MOL005970, MOL000358
53	DPP4	MOL013430, MOL013279, MOL013277, MOL011963, MOL009053, MOL007879, MOL005955, MOL005828, MOL005814, MOL002881, MOL002644, MOL002235, MOL001956, MOL001941, MOL001803, MOL000210, MOL000008, MOL000006
54	CYCS	MOL000008
55	CSF2	MOL000472
56	CREB1	MOL005828
57	CFLAR	MOL000008
58	CD40LG	MOL000008, MOL000006
59	CD163	MOL005828
60	CCND1	MOL000008, MOL000006
61	CAT	MOL004328
62	CASP9	MOL005828, MOL000358, MOL000008, MOL000006
63	CASP8	MOL000358
64	CASP3	MOL007930, MOL004328, MOL000472, MOL000471, MOL000358, MOL000008, MOL000006
65	BCL2L1	MOL000006, MOL000008
66	BCL2	MOL005828, MOL004328, MOL000358, MOL000008
67	BAX	MOL007930, MOL005828, MOL000471, MOL000358, MOL000008
68	BAD	MOL004328, MOL000008
69	BACE1	MOL007879
70	APP	MOL000006
71	APOB	MOL004328
72	AKT1	MOL004328, MOL000008, MOL000006
73	AKR1B1	MOL002268, MOL000471
74	ADRB2	MOL013437, MOL013436, MOL013279, MOL013277, MOL011971, MOL007879, MOL005970, MOL005955, MOL005849, MOL005814, MOL002644, MOL001956, MOL001803, MOL000358, MOL000210
75	ADRA2C	MOL013279
76	ACTA2	MOL000472
77	ACHE	MOL013277, MOL009053, MOL007879, MOL005970, MOL005814, MOL004792, MOL001803
78	ABCC8	MOL013277

### PPI network construction

**Figure 4.**
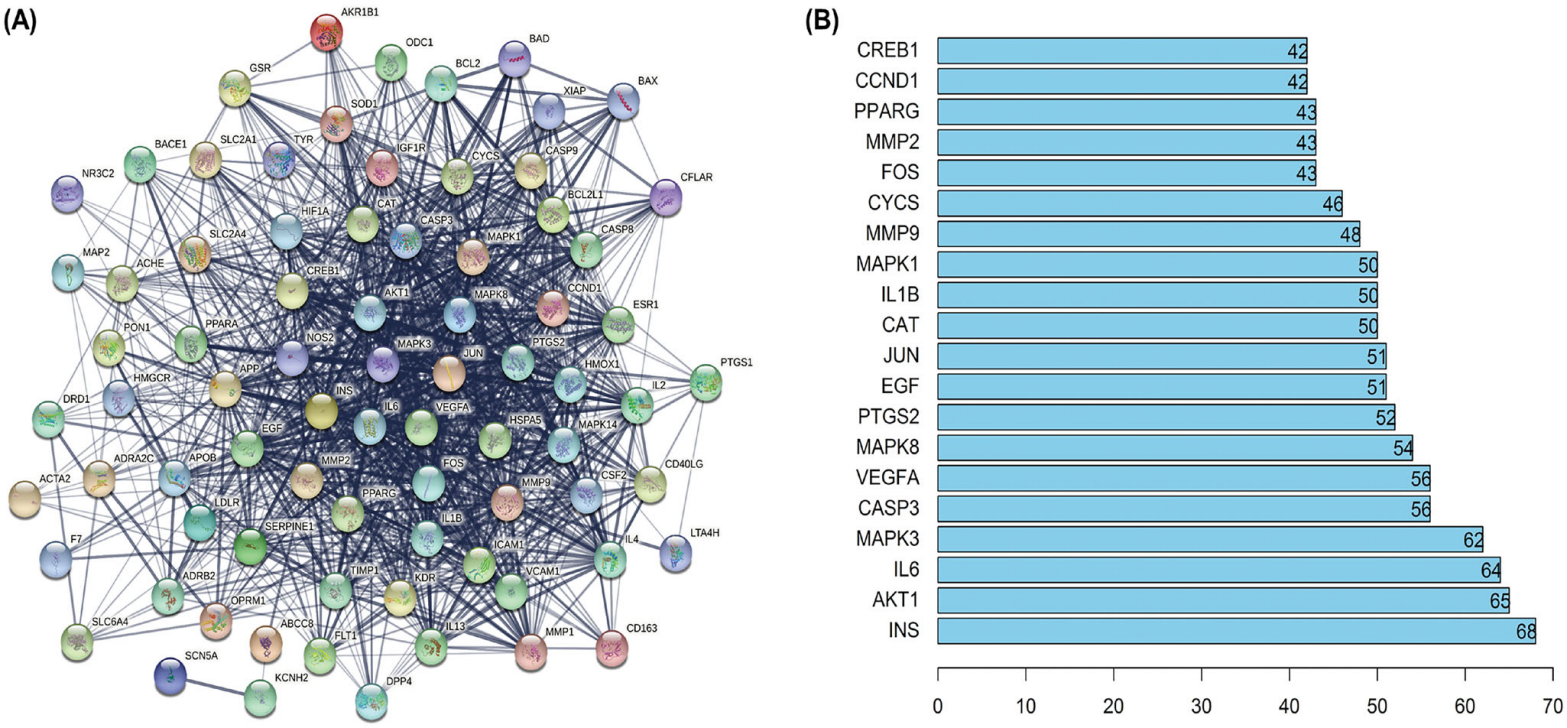
PPI networks of SHD treatment ischaemic stroke. (A) The network nodes represent proteins; the edge means line thickness indicates the strength of evidence. (B) Top 20 hub genes in the PPI network.

To elucidate the potential mechanism by which SHD protects against cerebral ischaemic stroke, PPI relationships of the 78 target genes were obtained using the STRING tool, and the results are displayed in Figure 4(A). There were 78 nodes and 1151 edges in the PPI network. Central network assessment is used to obtain key targets. As a key node to evaluate the entire network, hub targets are defined by their number of interactions. As shown in Figure 4(B), the target genes INS, Akt1, IL6, MAPK3, CASP3, VEGFA, MAPK8, PTGS2, EGF and JUN were likely the core targets in the network.

### Pathway and GO term enrichment analysis

**Figure 5.**
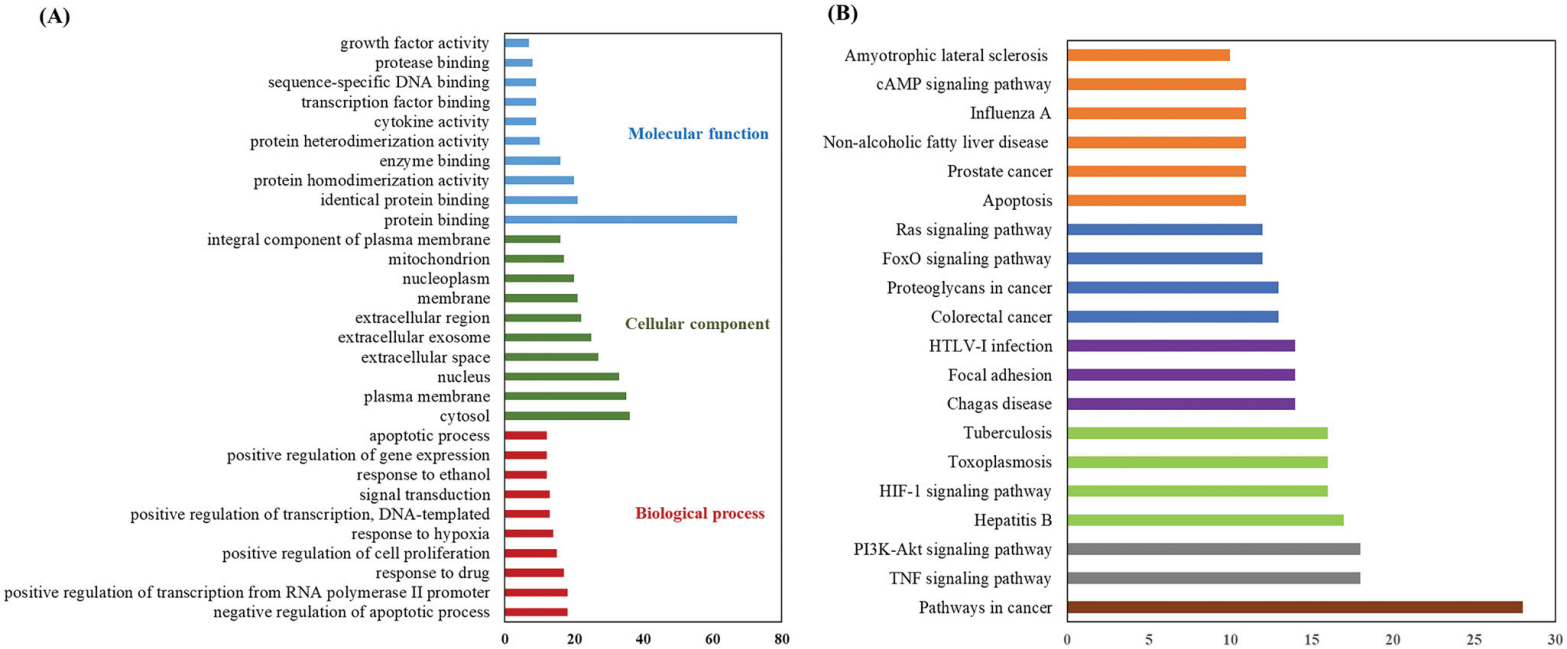
KEGG pathway and GO analysis using the DAVID database. (A) GO enrichment analysis. The red, green and blue colours represent biological process (BP), cellular component (CC) and molecular function (MF). (B) The chart of KEGG pathway enrichment.

GO and KEGG pathway analyses of 78 candidate targets were performed using the DAVID data. GO showed the BP, CC and MF associated with the targets. A total of 359 GO terms were considerably enriched, including 270 BP, 44 CC and 45 MF. The top 20 categories for BP, CC and MF are shown in Figure 5(A). Specifically, the targets were most highly enriched in the following biological process ontologies: negative regulation of apoptotic process, response to drug, and positive regulation of cell proliferation. The most highly enriched cellular component ontologies included cytosol, plasma membrane, nucleus, extracellular space and extracellular exosome. The synergistic effects of SHD included protein binding, isoprotein binding, enzyme binding and other molecular functions.

In addition, enrichment analysis of KEGG pathways showed that the 78 key targets were involved in 98 pathways. The top 20 pathways related to the pathogenesis of ischaemic stroke are shown in Figure 5(B).

### Molecular docking results

**Figure 6.**
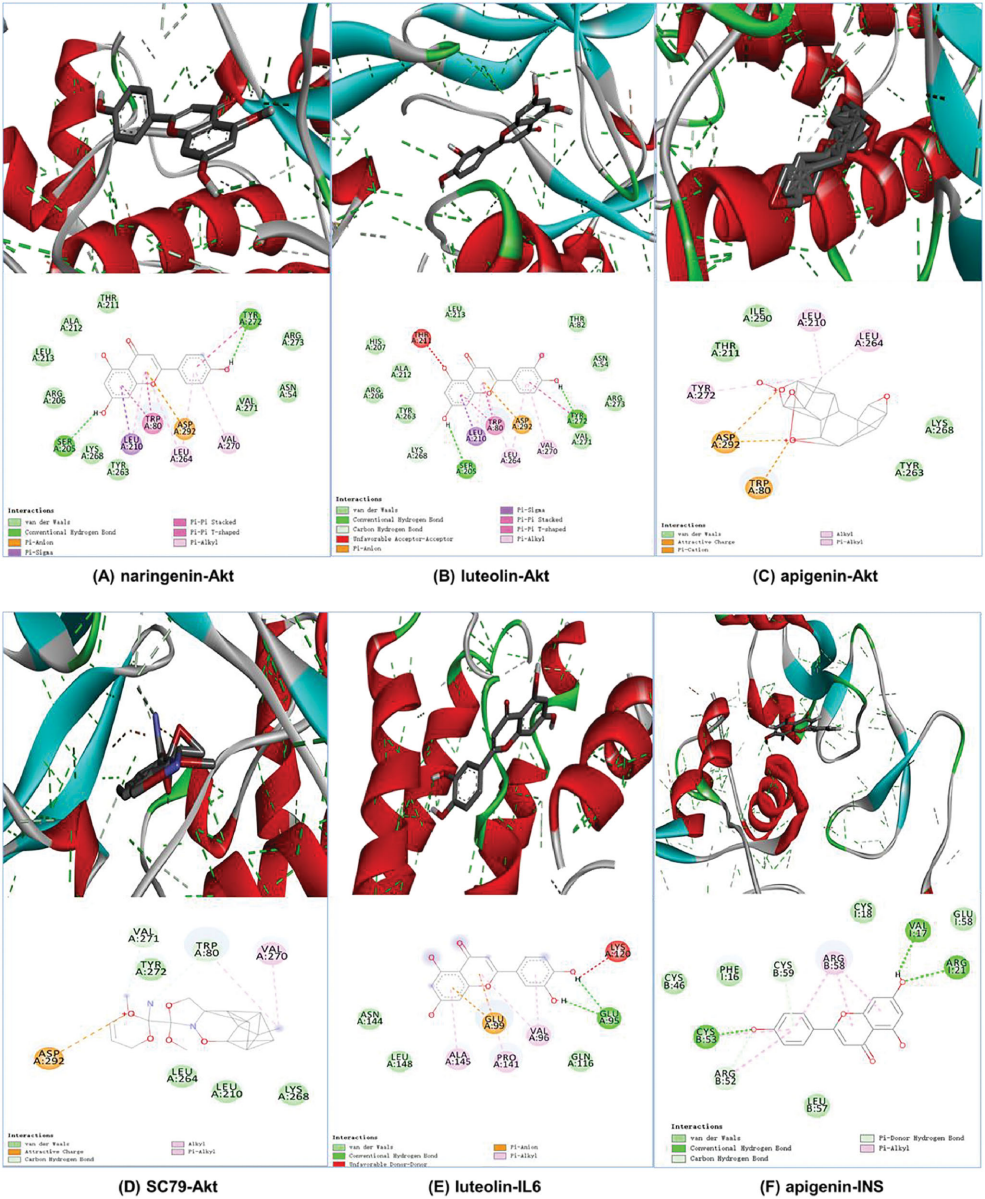
Molecular docking diagrams (3D and 2D). (A) Naringenin with Akt, (B) luteolin with Akt, (C) apigenin with Akt, (D) SC79 with Akt, (E) luteolin with IL6 and (F) apigenin with INS.

It is generally believed that ligand–receptor interactions with stronger affinities require less energy to interact and are more likely to interact (Lu et al. 2015; Cui et al. 2020). We selected the centrality value, centricity value and grade value of SHD in the ‘herb-compound-target’ network and comprehensively ranked the three active compounds (naringenin, luteolin and apigenin) their corresponding core targets (INS, Akt and IL-6) for docking analysis. The Akt agonist SC79 was used as a positive drug for molecular docking analysis. There was no suitable structure of the drug available for IL-6 and INS. The results revealed that these core component-targets had a strong binding activity. The affinity between these compounds and these three proteins was less than −5.0 kcal/mol. The docking results are shown in Table 3. The results demonstrated that naringenin (–10.2 kcal/mol) had better binding activity than the positive drug. Luteolin (–9.4 kcal/mol) and apigenin (–9.4 kcal/mol) had similar affinity with Akt, as well as the positive drugs, apigenin (–7.2 kcal/mol) had better binding activity with INS, and luteolin had a good affinity with IL-6. The docking results are shown in Figure 6.

**Table 3.** Binding energy values of the key ingredient in SHD with Akt, INS and IL6.

Ligand	CAS	Target	Affinity (kcal/mol)
SC79 (positive drug)	305834-79-1	Akt	–9.4
Naringenin	480-41-1	Akt	–10.2
Luteolin	491-70-3	Akt	–9.4
Apigenin	520-36-5	Akt	–9.4
Apigenin	520-36-5	INS	–7.2
Luteolin	491-70-3	IL6	–6.3

### SHD treatment alleviated OGD/R injury

**Figure 7.**
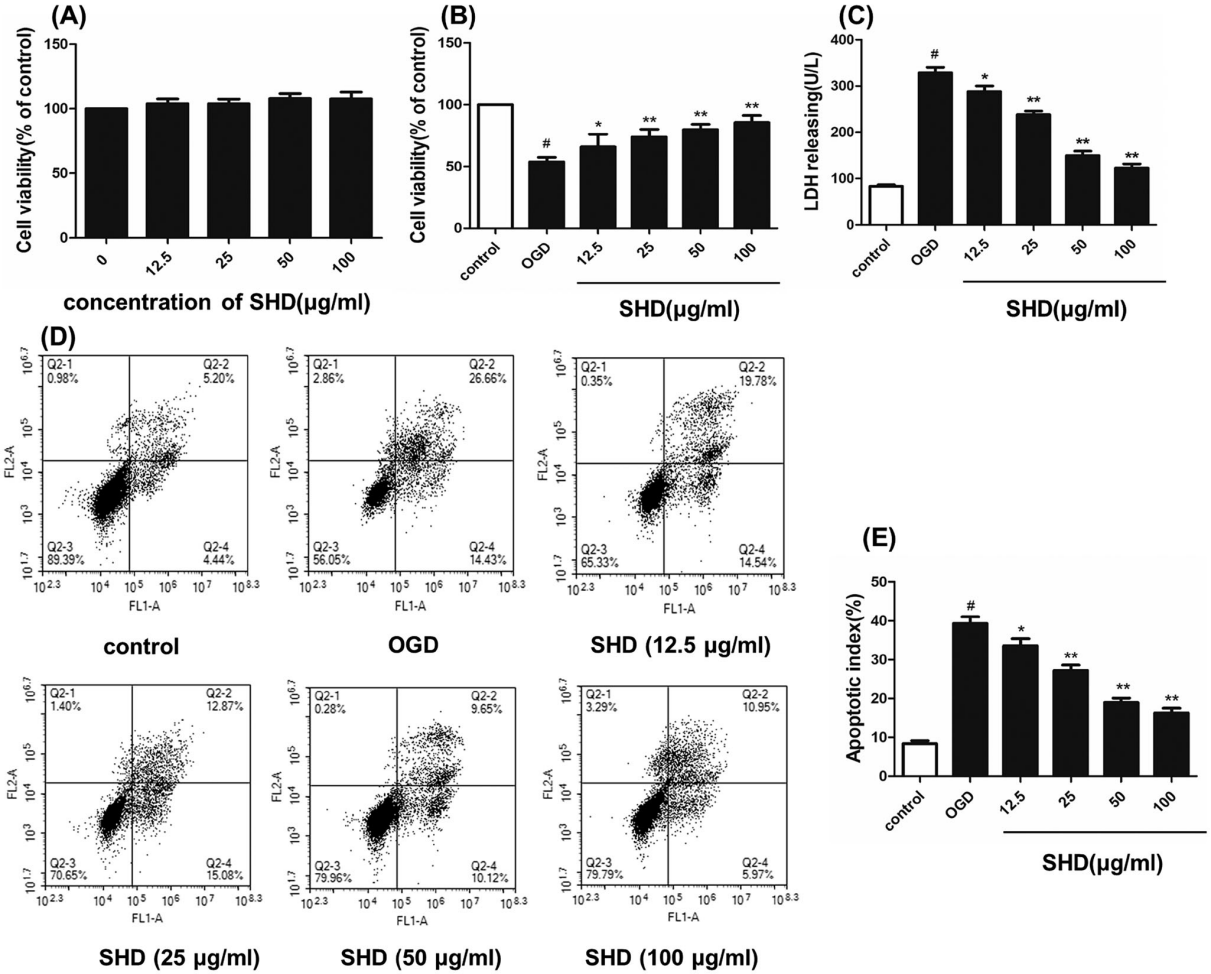
Protective effect of SHD against OGD/R injury. (A) The effect of SHD treatment on cell viability was assessed by MTT assay. (B) The effect of SHD treatment on cell viability subjected to OGD/R. (C) The lactate dehydrogenase (LDH) release levels. (D) Cell apoptosis was determined by Annexin V-FITC/PI staining. (E) Analysis of the apoptotic index. *n* = 6, ^#^*p* < 0.05, compared with the control group; *p < 0.05, **p < 0.01, compared with the OGD group.

SHD reduced ischaemia–reperfusion injury, as verified by experiments. As shown in Figure 7(A), SHD alone (12.5–100 μg/mL) showed slight effects on cell viability compared with the sham group. As shown in Figure 7(B), SH-SY5Y cell viability was significantly decreased in the OGD group (*p* < 0.05). However, pre-treatment with SHD (12.5, 25, 50 and 100 μg/mL) exhibited a protective effect and significantly improved the cell survival rate (*p* < 0.05). The protective effect of SHD was dose-dependent, and the median effective dose (ED_50_) value was 47.1 μg/mL. To further investigate the protective effect of SHD, LDH levels were measured. As shown in Figure 7(C), the OGD groups had distinctly increased LDH levels (*p* < 0.05). Conversely, SHD at different concentrations lowered LDH release (*p* < 0.05). As shown in Figure 7(D,E), the proportion of apoptotic cells in the OGD group was apparently increased compared with that in the sham group (*p* < 0.01). SHD treatment prevented the apoptosis induced by OGD (*p* < 0.01). Moreover, treatment with 100 μg/mL SHD elicited a greater protective effect against apoptosis than treatment with 12.5, 25 and 50 μg/mL SHD (*p* < 0.01). These findings confirmed the protective effect of SHD against neuronal cell damage.

### SHD prevented OGD/R injury by regulating the expression of p-PI3K, p-Akt, p-CREB, TNF-α and IL-6

**Figure 8.**
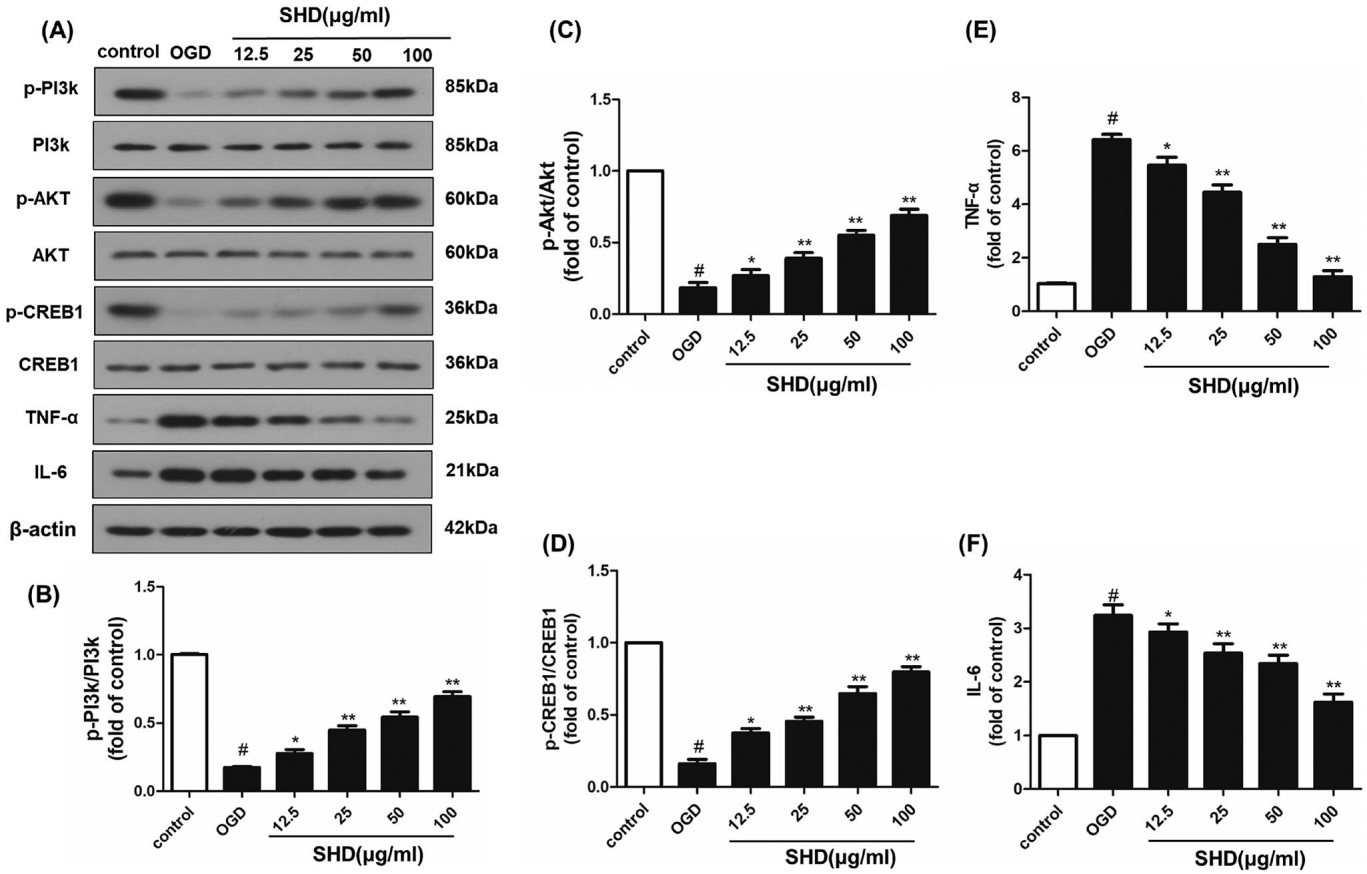
SHD promoted the phosphorylation of PI3k, Akt and CREB expression, and decreased TNF-α and IL6 expression. (A) The expression of p-PI3k, p-Akt, p-CREB, TNF-α and IL6 was detected by Western blotting. (B) SHD treatment increased the phosphorylation of PI3k. (C) SHD treatment increased the phosphorylation of Akt. (D) The expression of p-CREB1 was increased by treatment with SHD. (E) The expression of TNF-α was decreased by treatment with SHD. (F) SHD treatment mediated IL-6 expression compared with that in the untreated OGD group. *n* = 6, ^#^*p* < 0.05, compared with the control group; *p < 0.05, **p < 0.01, compared with the OGD group.

The network pharmacology results demonstrated that the potential targets of SHD against ischaemic stroke were significantly enriched in the PI3K-Akt and TNF pathways. The protein levels of PI3K, phosphorylated PI3K, Akt, phosphorylated Akt, TNF-α, IL-6, CREB1 and phosphorylated CREB1 were chosen for experimental validation. As shown in Figure 8, the p-PI3K, p-Akt and p-CREB1 levels in the OGD group were downregulated compared with those in the control group (*p* < 0.05), while the levels of Akt and CREB1 did not change. The OGD group showed significantly increased TNF-α and IL-6 expression (*p* < 0.05). SHD treatment distinctly improved the p-PI3K, p-Akt and p-CREB1 levels and reduced the overexpression of TNF-α and IL-6 compared with the OGD groups (Figure 8(B–F), *p < 0.05, **p < 0.01). These results demonstrated that the neuroprotective effect of SHD was likely mediated through the PI3K/Akt/CREB1 and TNF signalling pathways, as confirmed by network analysis.

## Discussion

Ischaemic stroke involves disordered metabolism or function caused by narrowing or occlusion of the cerebral arteries that supply blood to the brain. In the theory of TCM, ischaemic stroke is caused by sweat pore blocking and the obstruction of qi and blood (Zheng and Huang 2007). SHD alleviated ischaemic stroke injury by regulating qi and sweat pores. To clarify the beneficial effect of SHD on ischaemic stroke, we performed a series of experiments in combination with network pharmacology methods to confirm its effectiveness and molecular mechanism.

Sixty-seven constituents and 685 compound-related targets were verified in SHD, and 874 ischaemic stroke-related targets were confirmed from the database. Among these targets, 78 targets correlated with ischaemic stroke and SHD. According to the results of pathway enrichment, SHD may act against ischaemic stroke via the PI3K-Akt, TNF and HIF-1 pathways, among others. The results of the experiment demonstrated that SHD alleviated OGD-induced neuronal injury. Five key targets (PI3K, Akt, CREB1, TNF-α and IL-6) were validated through pharmacological experiments. By network pharmacological analysis, nine components (apigenin, luteolin, nobiletin, naringenin, beta-sitosterol, emodin, tetramethoxyluteolin, isosinensetin and tangeretin) with more than 10 target genes were identified as key components of the effect of SHD against ischaemic stroke. Apigenin improved neuronal survival through a molecular mechanism involving an increase in antioxidant and neurotrophic factors and a decrease in oxidants and proinflammatory molecules (Nabavi et al. 2018). Apigenin activated the caveolin1/VEGF pathway, alleviated neuronal apoptosis and autophagy and reduced brain injury (Pang et al. 2018). The potential mechanism of luteolin treatment ischaemic stroke involves regulating MMP9 and activating the PI3K/Akt pathway (Luo et al. 2019). Nobiletin attenuated brain ischaemic injury may be caused by improved permeability of the blood brain barrier through activating the Akt/CREB pathway (Zhang et al. 2013). Moreover, nobiletin inhibited the TLR4/NF-κB pathway by reducing proinflammatory cytokines (TNF-α, IL-1β and IL-6) and nitric oxide and improved propofol-induced neuroprotection (Zheng et al. 2017). Naringenin plays a neuroprotective role by promoting the proliferation of neurons, inhibiting cell apoptosis and oxidative stress, and regulating the nuclear translocation of the Nrf2 protein (Wang et al. 2017). Emodin has neuroprotective effects against ischaemia/reperfusion injury that may occur through activating the ERK-1/2 signalling pathway (Leung et al. 2020). The PI3K-Akt pathway is a crucial pathway in the pathological mechanism of ischaemic brain injury (Samakova et al. 2019). This signalling pathway regulates many cell functions, such as cell survival, autophagy, protein synthesis and glycolysis (Xie et al. 2019). The phosphorylation of Akt promotes the antiapoptotic protein Bcl2 and thus reduces cell apoptosis. Recent studies reported that Akt phosphorylation improved the nuclear translocation of Nrf2 and phosphorylation of the survival regulatory protein CREB and protected against brain ischaemia injury (Zhang et al. 2018).

In this study, our experiments verified that SHD increased the phosphorylation of PI3K, Akt and CREB1. Network pharmacological analysis indicated that the TNF signalling pathway is likely the second most important pathway for SHD protection against ischaemic brain injury. The study by Boehme et al. (2016) demonstrated that proinflammatory cytokines (such as IL-6 and TNF-α) were biomarkers of the recurrence of vascular events. The release of IL-6 after ischaemic stroke induces brain injury and hippocampal neuron necrosis by activating the NMDA receptor and upregulating JNK (Armstead et al. 2019). Moreover, the results showed that treatment with SHD extract reduced the expression level of TNF-α and IL-6, indicating that SHD attenuated ischaemic brain injury by inhibiting the expression of TNF-α and IL-6. The HIF-1 pathway is another crucial pathway for SHD in ischaemic stroke. HIF-1 is a transcription factor that plays a key role in the transcriptional adaptation of cells to hypoxia (Davis et al. 2018). Numerous studies have shown that activation of HIF-1 may be a potential therapeutic strategy for achieving significant neuroprotection after ischaemic stroke (Karuppagounder and Ratan 2012). Protection against ischaemic brain injury via SHD-mediated regulation of the HIF-1 signalling pathway should be confirmed in further studies.

## Conclusions

In the present study, network pharmacology analysis and experimental confirmation were performed to reveal the mechanisms underlying the protective effects of SHD against brain I/R injury. Network analysis of SHD identified 67 active compounds and 78 targets related to ischaemic stroke. Pathway enrichment demonstrated that SHD may protect against ischaemic stroke via the PI3K-Akt pathway, TNF pathway, HIF-1 pathway, etc. Western blot assays confirmed that the neuroprotective effect of SHD against ischaemic injury may be mediated via regulation of PI3K, Akt, CREB1, TNF-α and IL-6. This study provides a theoretical basis for further mechanistic studies of SHD against ischaemic stroke and demonstrates a breakthrough in elucidating the pharmacodynamic substance basis and mechanism of TCM in the treatment of ischaemic stroke.

## Authors contributions

Wei Zhang, Li Zhang and Wen jun Wang wrote the original manuscript and performed experiments. Shanbo Ma and Mingming Wang collected and analysed the data. Minna Yao, Ruili Li and Wei wei Li completed the literature research and molecular docking experiments, and Xian Zhao produced the chart of the experimental results. Dongmei Hu assisted with pharmacological experiments. Yi Ding and Jingwen Wang designed the experiments and provided overall coordination.

## Supplementary Material

Supplemental Material
