# Supplementary material for: Network pharmacology and in vitro experimental verification to explore the mechanism of Sanhua decoction in the treatment of ischaemic stroke
Source: Pharm Biol. 2022 Jan 5;60(1):119–30. doi: 10.1080/13880209.2021.2019281 (PMC8741256; doi:10.1080/13880209.2021.2019281)
Supplement: Supplemental Material [file IPHB_A_2019281_SM7931.docx]

**The quality control of Sanhua decoction by High performance liquid chromatography (HPLC)**

**Materials and methods**

The reference standards of gallic acid, syringin, naringin, hesperidin and neohesperidin (purity ≥ 98%) were purchased from Baoji Chenguang Biotechnology Co. Ltd. The acetonitrile, phosphoric acid and methanol used for HPLC-grade were purchased from Fisher Scientific (Fair Lawn, NJ, United States). Milli-Q water was prepared using a Milli-Q system (Millipore, MA, USA). The qualitative analysis of SHD was performed by LC-20AT HPLC system (Shimadzu, Kyoto, Japan) with an Agilent TC-C18 column (4.6 × 250 mm, 5 μm). The SHD powder 0.3 g dissolved in 25 mL methanol in a volumetric flask for qualitative analysis. The mixture of 5 reference standards were dissolved in methanol to prepare solutions with appropriate concentrations. The samples were filtered through 0.45 μm organic filter membrane, and 10 μL filtrate was injected into the HPLC-DAD system for analysis. The flow rate was 1 ml/min, the column temperature was 30°C. The mobile phase was composed 0.1% phosphoric acid in water (solvent A) and acetonitrile (solvent B) and the gradient elution procedure as follows: 0-8 min, 10% B; 8-25 min, 10%-25% B; 25-30 min, 25-45% B; 30-40 min, 45%-50% B; 45–50 min, 50-55% B; the UV detection wavelength was set at 270 nm.

**Results**

A total of 5 chemical constituents contained in the SHD were quantitatively determined using the HPLC method. The retention times of gallic acid, syringin, naringin, hesperidin and neohesperidin were 5.2, 13.6, 30.8, 31.5, and 32.4 min, respectively. The result was shown in Figure S1, all of the compounds detected could be chromatographically separated without interferences.


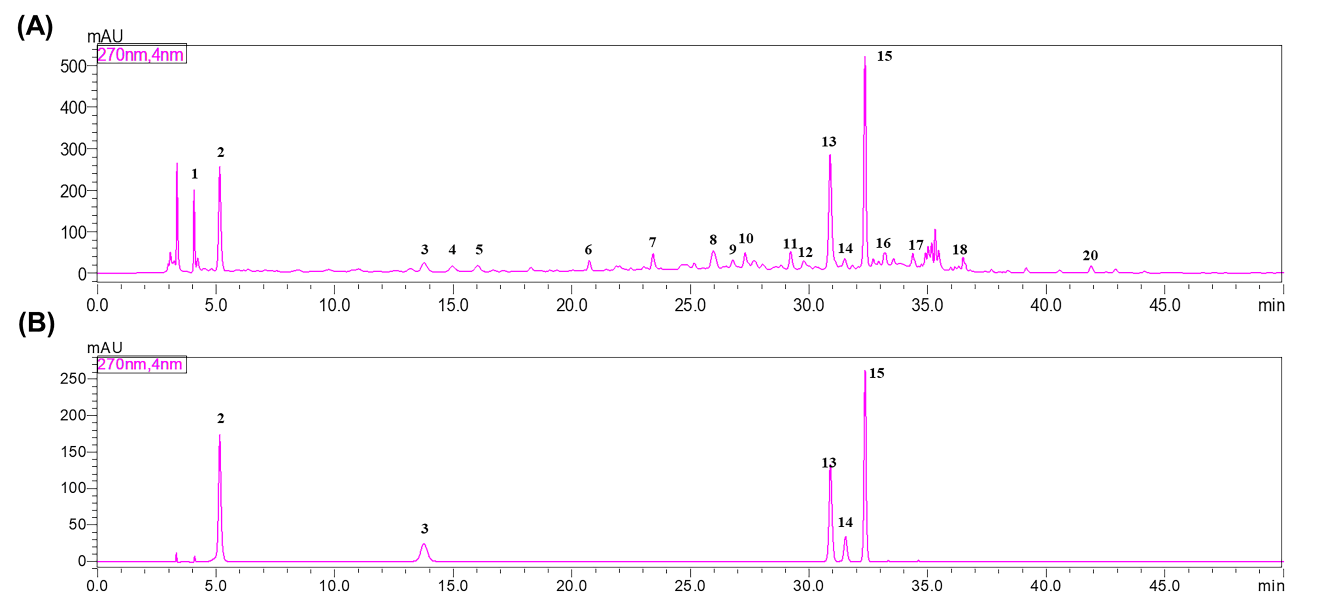


Figure S1. HPLC chromatograms of SHD (A) and mixed standard sample (B) at 270 nm. (2) Gallic acid, (3) Syringin, (13) Naringin, (14) Hesperidin, (15) Neohesperidin.
